# Predicting Adverse Perinatal Outcomes in Dichorionic Twin Pregnancies: A Multicentre Cohort Study

**DOI:** 10.1111/1471-0528.18125

**Published:** 2025-03-07

**Authors:** Veronica Giorgione, Mariarita Trapani, Miriam Lopian, Mariafrancesca Brutto, Maria Giulia Ferrante, Amarnath Bhide, Jacques C. Jani, Dominique A. Badr, Tullio Ghi, Basky Thilaganathan, Alessandra Familiari, Elisa Bevilacqua

**Affiliations:** ^1^ Fetal Medicine Unit, St George's University Hospitals NHS Foundation Trust London UK; ^2^ Department of Women and Child Health Women Health Area, Fondazione Policlinico Universitario Agostino Gemelli IRCCS Rome Italy; ^3^ Vascular Biology Research Centre, Molecular and Clinical Sciences Research Institute, St George's University of London London UK; ^4^ Department of Obstetrics and Gynecology University Hospital Brugmann, Université Libre de Bruxelles Brussels Belgium; ^5^ Catholic University of the Sacred Heart Rome Italy

**Keywords:** adverse perinatal outcome, estimated fetal weight, fetal growth restriction, intertwin discordance, intrauterine demise, middle cerebral artery, multiple pregnancy, stillbirths, twin pregnancies, umbilical artery

## Abstract

**Objective:**

Dichorionic twin pregnancies are associated with increased risks of stillbirth or medically indicated early preterm birth (ePTB) to avoid stillbirths. This study evaluated the predictive value of fetal estimated weight (EFW) and Doppler indices before adverse perinatal outcomes.

**Design:**

Retrospective multicentre cohort study.

**Setting:**

Three tertiary centres in the UK, Italy and Belgium.

**Population:**

The study included 1294 dichorionic twin pregnancies managed between 2013 and 2023.

**Methods:**

Univariable and multivariable analyses assessed the association and the predictive accuracy between EFW and Doppler indices taken within 2 weeks of birth or adverse perinatal outcomes.

**Main Outcome Measures:**

Stillbirths (of one or both twins) or medically indicated ePTB before 34 weeks' gestation for fetal indications.

**Results:**

The study identified 58 pregnancies (4.5%) complicated by adverse perinatal outcomes. There were significant differences (all *p* < 0.001) between twins with adverse perinatal outcomes and liveborn twins for small for gestational age foetuses (89.5% vs. 59.3%), EFW discordance (31.8% vs. 8.4%), umbilical artery (UA) pulsatility index (PI) discordance (39.7% vs. 12.6%) and middle cerebral artery PI discordance (27.6% vs. 13.3%). These associations remained significant after adjusting for maternal characteristics and gestational age. The best predictive model included EFW discordance and UA PI discordance, with an area under the curve of 0.90.

**Conclusions:**

The integration of intertwin EFW and UA PI discordance can effectively predict stillbirths or the need for medically indicated ePTB. After external validation in larger populations, this model could provide effective risk stratification of dichorionic pregnancies to enable targeted interventions to improve clinical outcomes.

## 1 Introduction

Dichorionic twin pregnancies account for two‐thirds of twin pregnancies and carry an elevated risk of adverse perinatal outcomes compared to singleton pregnancies, with increased rates of early preterm birth and perinatal deaths [1, 2]. These risks are most frequently due to uteroplacental insufficiency, which results in disparities in growth patterns between twins, creating challenges for the clinical management of the entire pregnancy [3]. Accurate risk assessment is essential in dichorionic twin pregnancies to guide prenatal management and improve perinatal outcomes.

In current clinical practice, the most used predictors of adverse outcomes in twin pregnancies are fetal size and fetal Doppler indices [4, 5, 6, 7]. As in singleton pregnancy, these parameters provide important insights into uteroplacental function and fetal wellbeing. While estimating twin fetal size centile is complicated by the current debate over the use of twin versus singleton growth reference charts [8, 9, 10], unique to twin pregnancy and independent of this debate is the ability to evaluate inter‐twin estimated fetal weight (EFW) size discordance. Inter‐twin size discordance relies on using the larger twin as a control, and elevated EFW discordance is associated with impaired uteroplacental function and is an independent predictor of twin mortality and morbidity [6, 11]. Similarly, fetal Doppler indices in the umbilical artery (UA) and middle cerebral artery (MCA) offer non‐invasive methods for identifying circulatory changes that have been shown to indicate fetal compromise in singleton and twin pregnancies. As with fetal size, the use of UA and MCA Doppler PI discordance overcomes the yet unsettled debate over the correct reference standard to be used [7].

There are some studies looking at the individual impact of EFW and fetal Doppler on adverse perinatal outcomes in dichorionic twin pregnancies complicated by selective fetal growth restriction (SFR) [3, 4, 12]. However, an evaluation of the combined value of EFW and fetal Doppler indices, either as individual centiles or as intertwin discordance, has never been undertaken to predict adverse outcomes [3, 4, 7]. This multicentre study aims to address this gap by developing predictive models for adverse perinatal outcomes using a range of fetal weight and fetal Doppler parameters.

## 2 Methods

This study was a retrospective multicentre cohort study conducted at three tertiary centres specialised in fetal medicine in the UK (St George's Hospital, London), Italy (Fondazione Policlinico Universitario Agostino Gemelli IRCCS, Rome) and Belgium (University Hospital Brugmann, Brussels). The study included twin pregnancies managed in the three centres between 2013 and 2023 that met the inclusion criteria.

Included patients were dichorionic pregnancies with known birth outcomes that underwent an obstetric ultrasound within 2 weeks of the birth or the diagnosis of an intrauterine death of one or both foetuses. In cases where one twin experienced stillbirth and the other survived, we ensured that the ultrasound data analysed corresponded to the foetus‐specific outcomes by using measurements obtained before the adverse event, thereby attributing predictive parameters accurately to each twin. Exclusion criteria included pregnancies complicated by structural anomalies, chromosomal abnormalities, early and late miscarriage (up to 21 + 6), or monochorionic twin pregnancies. In this study, we defined late miscarriage as pregnancy loss occurring before 22 weeks of gestation. This cut‐off was chosen to encompass international differences, as definitions of late miscarriage vary across countries, typically ranging between 20 and 24 weeks. Chorionicity and gestational age were confirmed by ultrasound assessment performed in the first trimester [13]. Country‐specific research approvals or research exemptions were obtained for the use and retrospective analysis of routinely collected anonymised data. This manuscript adheres to the TRIPOD guidelines, ensuring comprehensive and transparent reporting of prediction model development and validation. Patients were not involved in the design, conduct, reporting, or dissemination of this research.

Patients were identified through a retrospective search of ultrasound databases, and data regarding maternal demographics, pregnancy, and their outcomes were collected from the same ultrasound databases and electronic medical records. In all three centres, the frequency of ultrasound assessments during pregnancy and delivery timing followed ISUOG and FIGO international guidelines [13, 14]. Twins were labelled based on EFW or birthweight as twin 1 for the smaller and twin 2 for the larger twin. EFW was calculated using the Hadlock formula based on head circumference, abdominal circumference, and femur length, while EFW centile was calculated from singleton standards established by the Fetal Medicine Foundation [15, 16]. Small for gestational age (SGA) were defined as EFW below the 10th, 5th, or 3rd centile. EFW discordance was calculated from the formula (EFW larger twin−EFW smaller twin)/EFW larger twin × 100. Doppler indices, including the umbilical artery (UA) pulsatility index (PI) and middle cerebral artery (MCA) PI, were recorded in the last ultrasound, and discordance in UA and MCA measurements between twins was calculated as UA PI discordance = (Higher UA PI−Lower UA PI)/Higher UA PI × 100% and MCA PI discordance = (Higher MCA PI−Lower MCA PI)/Higher MCA PI × 100. Cerebroplacental ratio (CPR) was also calculated as MCA PI/UA PI, and CPR discordance = (Higher CPR−Lower CPR)/Higher CPR × 100. UA, MCA, and CPR centile were calculated using standards established by the Fetal Medicine Foundation [17].

The outcome was stillbirth and medically indicated early preterm birth (ePTB) for fetal indications, which included abnormal ultrasound, Doppler, or CTG findings. This definition was chosen to encompass both actual and potential stillbirths, as pregnancies delivered preterm due to severe fetal compromise are at significant risk of intrauterine demise if left undelivered. Stillbirth was defined as intrauterine death after 22 weeks' gestation, and ePTB was defined as delivery before 34 weeks' gestation. Neonatal death is defined as the death of a live‐born infant occurring within the first 28 days of life. Spontaneous and medically indicated PTB were further characterised based on the aetiology of the premature birth.

### 2.1 Statistical Analysis

Variables were assessed for normality by visualising their histograms, and decisions regarding the functional form of variables were guided by clinical and biological understanding of the parameters. Data from categorical variables were expressed as *N* (%) and from continuous variables as median and interquartile range (IQR). Categorical data were compared using the Chi‐square test, and continuous variables were analysed using the Mann–Whitney U‐test. The sample size for this study was determined based on the total number of eligible cases available in the datasets from the three participating centres rather than formal sample size calculations. The study included 58 outcome events (4.5% of the cohort), which are consistent with recommendations for a sufficient number of events per predictor variable when developing prediction models [18]. To minimise the risk of overfitting, the number of predictors included in the final models was deliberately limited to a maximum of three variables. Missing data were handled using a complete‐case analysis approach, given the low proportion of missing data for key variables such as EFW and UA PI. Cases with absent outcome data or missing ultrasound information prior to the outcome of interest were excluded from inception by the three centres. Binomial logistic regression models were used to evaluate the association between maternal factors, EFW discordance, SGA of at least one twin, UA PI discordance and MCA PI discordance and the outcome of interest (stillbirth and medically indicated preterm delivery before 34 weeks for fetal indications). Multivariable models were also undertaken to compare if differences in fetal factors between cohorts persisted after adjusting for maternal age, non‐white ethnicity, BMI, and gestational age at ultrasound. Variables included in the final prediction models were selected based on their statistical significance in univariate analyses. Despite the skewness of continuous data (EFW discordance and UA PI discordance), model performance was superior with the original variables, so data transformation of these variables was unnecessary. Multicollinearity was assessed using Variance Inflation Factor (VIF) and Tolerance values, with VIF < 5 and Tolerance > 0.2 considered acceptable. Receiver Operating Characteristic (ROC) curves were performed to examine the efficacy of variables in detecting pregnancies with the outcome, and the relative results were reported as the area under the curve (AUC) and 95% confidence interval (95% CI). Calibration tests (such as Hosmer–Lemeshow Test), model intercepts, and coefficients were reported in the Supporting Information for all the models (Table S1). Internal validation was performed using bootstrapping with 1000 resamples to evaluate the model's performance and adjust for potential optimism and overfitting. Screening performance of EFW discordance (> 25%, > 20% and> 15%) and SGA defined as EFW < 10th, 5th, and 3rd centile was evaluated with sensibility and specificity. The statistical analysis was performed using SPSS 28.0 (SPSS Inc., Chicago, IL, USA), with statistical significance set at a *p* value of < 0.05.

## 3 Results

The study cohort consisted of 1294 dichorionic twin pregnancies (Figure S1) from Italy (*n* = 367), Belgium (*n* = 255) and the UK (*n* = 672). Early PTB < 34 weeks' gestation was observed in 16.3% (211/1294) of the pregnancies (9.7% spontaneous ePTB and 5.8% medically indicated ePTB). Medically indicated ePTB for fetal reasons occurred in 3.32% (43/1294) of cases. The median (IQR) gestational age at delivery was 36.71 (34.86–37.29) and birthweight centile (IQR) was 2.42 (0.28–10.76) for the smaller twin (twin 1) and 18.14 (5.52–37.46) for the larger twin. There were 58 (4.5%) pregnancies that met the study outcome criteria complicated by at least one stillbirth (*n* = 15) or medically indicated PTB for fetal indications (*n* = 43). The median (IQR) interval in days between the last ultrasound assessment and the outcome of interest was 8 [4, 5, 6, 7, 8, 9, 10, 11]. Stillbirth happened in 1.1% (*n* = 14) of the first twin and 0.4% (*n* = 5) of the second twin, while neonatal deaths occurred in 1.2% (*n* = 15) and 0.7% (*n* = 9), respectively. Double perinatal loss, including stillbirth or neonatal deaths, complicated 14 dichorionic pregnancies (1.1%).

Univariate analysis revealed significant associations for variables such as higher EFW discordance, SGA proportions, UA PI discordance, and MCA PI discordance with the study outcome of stillbirth or indicated ePTB for fetal concerns (Table 1). EFW discordance median (IQR) was markedly higher among cases with adverse outcomes (31.75%, 12.81% to 47.15%) compared to controls (8.35%, 3.82% to 14.63%, *p* < 0.001). The findings were similarly significant for SGA < 10th centile, SGA < 3rd centile, as well as UA PI, MCA PI, and CPR discordances (Table 2). These associations and odds ratios (ORs) remained significant when adjusted (aORs) for maternal age, non‐white ethnicity, BMI, and gestational age at ultrasound assessment.

**TABLE 1:** Univariate and multivariate analysis for associations between maternal and fetal variables with stillbirth (SB) or medically indicated early preterm birth (ePTB) for fetal reasons.

Variables	Livebirth (*n* = 1236)	SB or medically indicated ePTB (*n* = 58)	*p*	OR (95% CI)	*p*	aOR^a^ (95% CI)	*p*
**Maternal characteristics**							
Maternal age (years)	33 (29–37)	33 (30–37)	0.619	1.03 (0.98–1.07)	0.246	—	—
BMI (kg/m^2^)	25 (22–29)	25 (23–27)	0.855	0.98 (0.93–1.04)	0.525	—	—
Non‐white ethnicity	272 (25)	12 (22)	0.642	0.86 (0.44–1.65)	0.643	—	—
Smoking	61 (5)	2 (3.5)	1.00	0.70 (0.17–2.92)	0.620	—	—
Alcohol	19 (1.7)	1 (2)	0.586	1.18 (0.16–9.02)	0.871	—	—
IVF conception	267 (22.3)	12 (21.8)	0.940	0.98 (0.51–1.88)	0.940	—	—
Nulliparity	515 (47)	31 (60.8)	0.054	1.75 (0.98–3.11)	0.057	—	—
**Fetal weight parameters**							
EFW discordance	8.35 (3.82–14.63)	31.75 (12.81–47.15)	< 0.001	1.11 (1.08–1.13)	< 0.001	1.08 (1.06–1.11)	< 0.001
EFW discordance > 15%	295 (24.5)	42 (73.7)	< 0.001	8.62 (4.71–15.77)	< 0.001	9.16 (4.03–20.83)	< 0.001
EFW discordance > 20%	152 (12.6)	42 (73.7)	< 0.001	19.36 (10.48–35.76)	< 0.001	24.44 (9.92–60.21)	< 0.001
EFW discordance > 25%	72 (6.0)	21 (36.8)	< 0.001	26.93 (14.95–48.51)	< 0.001	28.33 (12.07–66.49)	< 0.001
Any SGA < 10th centile	714 (59.3)	51 (89.5)	< 0.001	5.83 (2.48–13.70)	< 0.001	6.80^b^ (2.40–19.30)	< 0.001
Any SGA < 3rd centile	524 (43.5)	51 (89.5%)	< 0.001	11.03 (4.70–25.90)	< 0.001	13.00^b^ (4.58–36.84)	< 0.001
**Fetal Doppler indices**							
UA PI discordance	12.64 (6.40–21.35)	39.65 (26.50–54.95)	< 0.001	1.09 (1.07–1.11)	< 0.001	1.09 (1.06–1.11)	< 0.001
Any UA PI > 95th centile	195 (16.5)	45 (86.5)	< 0.001	32.57 (14.48–73.29)	< 0.001	32.72 (13.53–79.16)^b^	< 0.001
Any UA PI AREDF	8 (0.7)	22 (40.7)	< 0.001	101.84 (42.15–246.03)	< 0.001	71.65 (27.18–188.89)	< 0.001
MCA PI discordance	13.35 (6.52–22.42)	27.64 (13.49–35.84)	< 0.001	1.03 (1.02–1.05)	< 0.001	1.03 (1.02–1.05)	< 0.001
CPR discordance	17.74 (8.68–30.39)	58.80 (35.75–70.82)	< 0.001	1.06 (1.05–1.08)	< 0.001	1.06 (1.04–1.08)	< 0.001

*Note:* Data are presented as *N* (%) or median (IQR). SB: stillbirth, Early PTB: preterm birth before 34 weeks' gestation.

Abbreviations: AR, absent or reverse; BMI, body mass index; CPR, cerebroplacental ratio; EDF, end diastolic flow; EFW, estimated fetal weight; MCA, middle cerebral artery; PI, pulsatility index; SGA, small for gestational age; UA, umbilical artery.

^a^ Adjusted for maternal age, BMI, non‐white ethnicity, and gestational age at ultrasound.

^b^ Not adjusted for gestational age at ultrasound as centiles are already corrected for it.

**TABLE 2:** Prediction models for stillbirth and/or medically indicated preterm birth before 34 weeks' gestation for fetal indications.

Models	Variables	OR	95% CI	AUC	95% CI
1	EFW discordance	1.07	1.04–1.10	0.90	0.84–0.95
	UA PI discordance	1.06	1.04–1.04		
2	EFW discordance	1.08	1.05–1.12	0.92	0.88–0.97
	UA PI discordance	1.07	1.05–1.10		
	MCA PI discordance	1.01	0.98–1.03		
3	SGA < 10th centile	3.97	1.37–11.52	0.88	0.82–0.94
	UA PI discordance	1.08	1.06–1.10		
4	SGA < 10th centile	4.25	1.25–14.50	0.90	0.85–0.95
	UA PI discordance	1.10	1.07–1.12		
	MCA PI discordance	1.01	1.00–1.03		

Abbreviations: AUC, area under the curve; CI, confidence interval; EFW, estimated fetal weight; MCA, middle cerebral artery; OR, odd ratio; PI, pulsatility index; SGA, small for gestational age; UA, umbilical artery.

**FIGURE 1:**
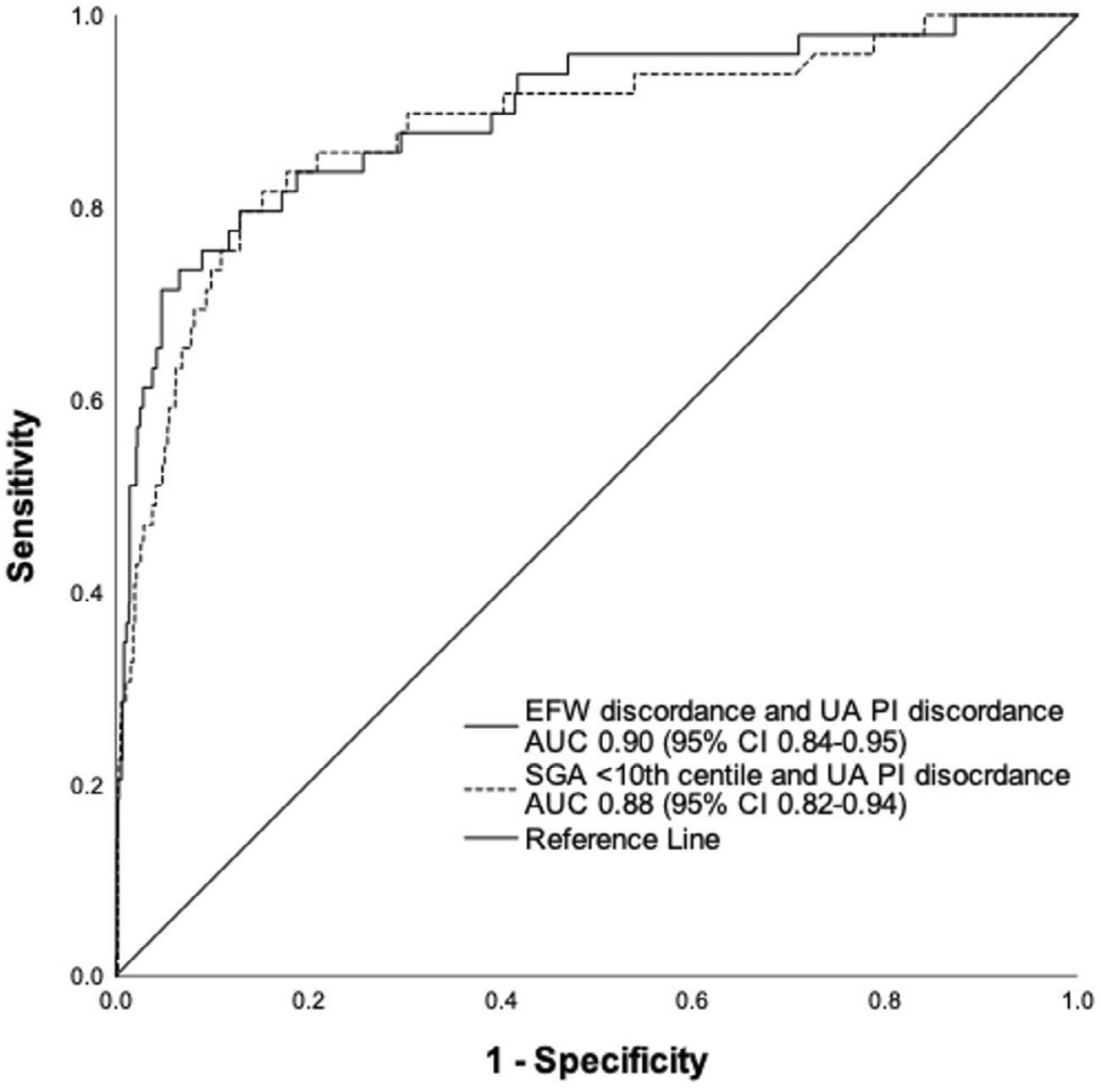
ROC curves for EFW discordance versus SGA < 10th centile when used with UA PI discordance to predict adverse perinatal outcomes. AUC, area under the curve; CI, confidence interval; EFW, estimated fetal weight; SGA, small for gestational age; UA PI, umbilical artery pulsatility index.

**FIGURE 2:**
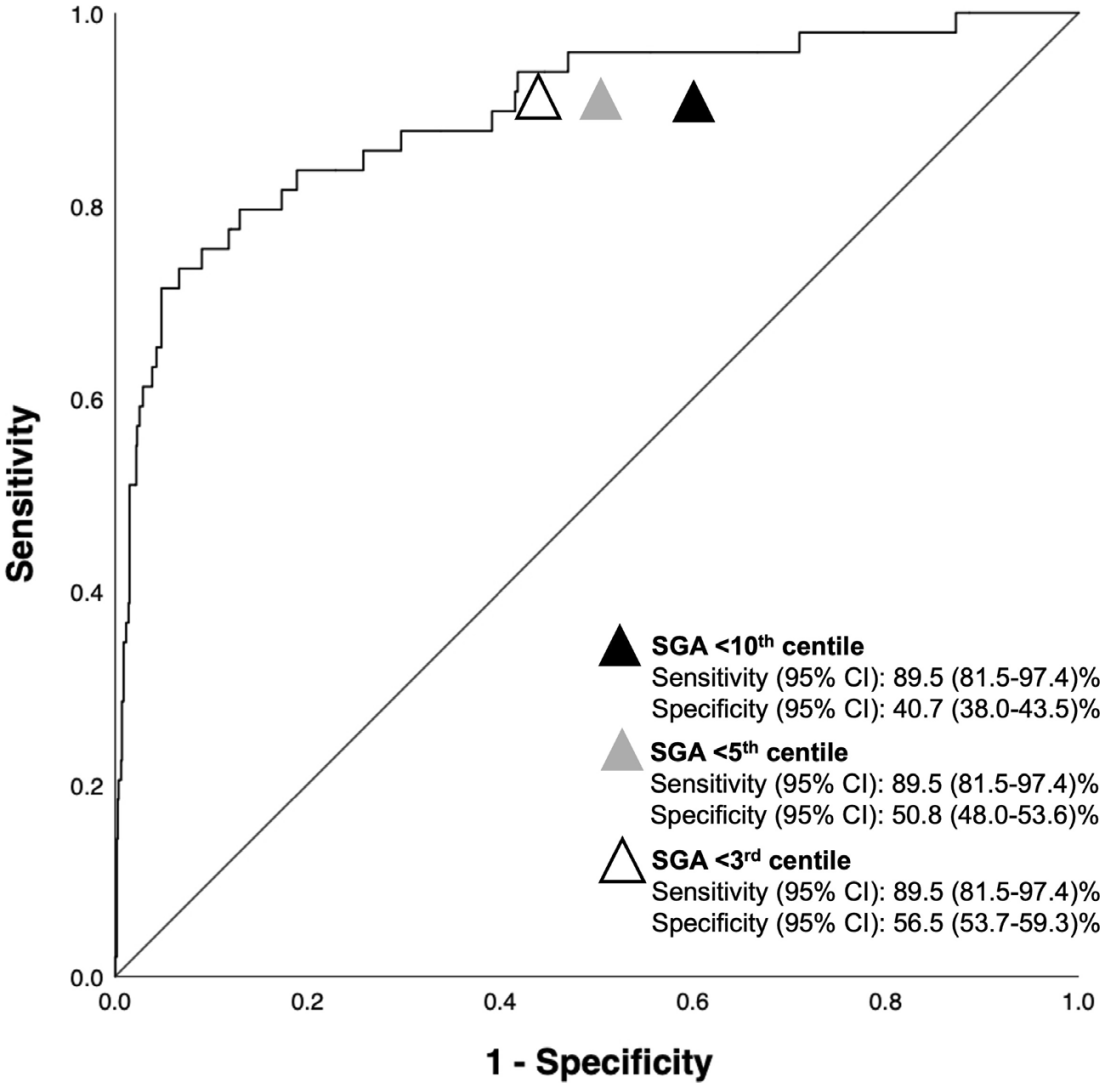
Sensitivity and specificity of the prediction model including EFW discordance and UA PI discordance versus SGA less than 10th centile, 5th centile, and 3rd centile. CI, confidence interval; EFW, estimated fetal weight; SGA; small for gestational age; UA PI, umbilical artery pulsatility index.

Prediction models using both fetal growth parameters and Doppler indices achieved excellent discriminatory power for the outcome of interest (Table 2, Table S1). The best model with the least number of variables was achieved using EFW discordance and UA PI discordance with AUC 0.90 (95% CI 0.84–0.95). Bootstrapped logistic regression confirmed the robustness of these predictors, with bias‐corrected and accelerated (BCa) confidence intervals remaining statistically significant (EFW discordance: BCa 95% CI: 1.043–1.094; UA discordance: BCa 95% CI: 1.043–1.084). Substituting SGA < 10th centile for EFW discordance in this model reduced the AUC to 0.88 (95% CI 0.82–0.94) (Figure 1). Similar results were obtained when neonatal deaths were added to the outcome (Table S2). The sensitivity and specificity of the model, including EFW discordance and UA PI discordance, were superior to that for set thresholds of EFW discordance > 15% (73.7%; 75.5%), EFW discordance > 20% (73.7%; 87.4%) and EFW discordance > 25% (63.2%; 94.0%, Figure S2). The findings were similar when the model was compared to SGA < 10th, < 5th, and 3rd centiles in place of EFW discordance as dichotomous variables (Figure 2).

## 4 Discussion

### 4.1 Main Findings

The findings of this study indicate that in dichorionic twin pregnancies, both fetal growth and haemodynamic parameters are valuable in predicting stillbirth or the need for scheduled birth to reduce the risk of perinatal loss. Higher EFW and UA PI discordances were significantly associated with an increased risk of an adverse pregnancy outcome, and a prediction model containing only these variables yielded an excellent AUC value (0.92). The study findings also demonstrate that a multifactorial assessment encompassing fetal size and haemodynamic inter‐twin discordances in dichorionic twin pregnancies is superior to the conventional cut‐offs used in clinical practice for either EFW discordance or SGA centile. These results support the potential for targeted clinical monitoring for adverse pregnancy outcomes in dichorionic twin pregnancies using a prediction model containing EFW and UA PI discordances as continuous rather than dichotomous variables.

### 4.2 Strengths and Limitations

A significant strength of this study is the large cohort of 1294 dichorionic twin pregnancies taken from three geographically distinct tertiary centres, which enhances the statistical power and generalisability of our findings. The study can more confidently identify associations between clinical factors and adverse outcomes by leveraging a large dataset. Another strength is that the recruiting hospitals are referral centres for twin pregnancies with standardised clinical protocols: the rate of adverse outcomes and the pathway of care were similar among the centres, with a limited risk of bias. Despite this, before this prediction model is introduced into clinical practice, it is essential that it undergoes external validation in an independent cohort to assess its generalisability and performance in different populations. We plan to collaborate with other major fetal medicine centres to apply the model to datasets from these institutions. This external validation process will involve recalibrating the model, if necessary, to ensure its accuracy and reliability across varying clinical settings. A general concern about such retrospective studies is bias because of population selection and clinical intervention bias. Furthermore, even though the inclusion of multiple clinical centres increases the generalisability of the findings, variations in clinical practice may have impacted data consistency. Finally, whilst we accounted for several demographic and clinical factors, other unmeasured confounders might influence outcomes and warrant further investigations. Data on hypertensive disorders of pregnancy or other pre‐existing maternal conditions, such as chronic hypertension or diabetes, that could have affected the uteroplacental function were not included in the data collection; these factors will be further investigated in future research. We acknowledge that the use of a composite outcome combining stillbirth and ePTB for fetal indications may not fully differentiate between the severities of these outcomes. Future studies with larger sample sizes could stratify these outcomes and develop separate predictive models for stillbirth and ePTB.

### 4.3 Interpretation

Dichorionic twin pregnancies are often managed similarly to singleton pregnancies, as recommended by most international guidelines [3, 19]. Despite this, they still carry an elevated risk of stillbirth or scheduled preterm birth due to a presumed increased risk of perinatal loss [1, 3, 20, 21]. Thus, there is an urgent need for data to guide targeted clinical management and timing of birth in dichorionic pregnancies, and the findings of our study represent an initial step towards providing such guidance. Our results demonstrate that EFW discordance as a continuous rather than a dichotomous variable is more clinically useful in assessing uteroplacental insufficiency and its consequences in dichorionic twin pregnancies. Similarly, discordance in fetal Doppler indices provides additional clinically useful information in the prediction of adverse pregnancy outcomes in these pregnancies.

Classifying one twin as SGA based on the < 10th percentile cut‐off does not account for intertwin differences and may lead to misclassification of the smaller twin, which is more likely to be constitutionally small rather than complicated by placental dysfunction. Furthermore, there is an ongoing debate regarding which growth charts—singleton or twin‐specific—should be used to determine EFW centiles and define SGA in twin pregnancies [8, 9, 10, 22]. While singleton charts may over‐diagnose growth restriction due to the physiological differences in twin growth trajectories, twin‐specific charts may risk underestimating true pathological growth restriction, as twin growth slows relative to singletons in late gestation [8, 9, 10, 22]. The latter debate is undermined by the lack of data relating the EFW centile to pregnancy outcome data. The association between UA PI centiles and adverse perinatal outcomes in dichorionic twins complicated by sFGR is well established [4, 19]. However, there are only very limited data from small studies on the individual predictive value of EFW and Doppler indices for adverse outcomes, with one study suggesting scheduled birth between 32 and 34 weeks for increased UA PI even with positive end diastolic flow in pregnancies complicated by sFGR [3, 4, 7].

In contrast, both inter‐twin EFW and Doppler biometric discordances capture the relative difference between the smaller twin and its larger ‘control’ co‐twin [12]. This metric is independent of the choice of fetal growth chart and provides insights into discordant placental function, which has a dose–response relationship with adverse perinatal outcomes in dichorionic pregnancies [23, 24, 25, 26]. The latter seems to be better at predicting adverse outcomes than the dichotomous discordance cut‐offs for predicting adverse outcomes endorsed by current guidelines recommending 25% EFW discordance independent of gestation, even though the most appropriate cut‐off has yet to be definitively established [13, 27, 28].

## 5 Conclusion

Dichorionic twin pregnancies follow a well‐defined protocol of antenatal surveillance based on monthly fetal growth assessments, as this strategy resulted in the early identification of twins at high risk of adverse outcomes [13]. Subsequent clinical management relies on EFW discordance cut‐off and fetal Doppler values, which are often biased by the chosen reference chart and do not take into account the relative difference between the twins. Poor pregnancy outcomes are mainly a consequence of discrepancies in risk assessment and inconsistency in the management pathway because of ineffective identification of those foetuses with a real risk of placental insufficiency and stillbirth. Targeted interventions are pivotal in order to improve perinatal outcomes for these high‐risk pregnancies not only to reduce the rate of stillbirth possibly but also to discriminate constitutionally small babies that can reach and go beyond 32 weeks of gestation, reducing, then, the rate of unnecessary premature birth. Our results show that this is feasible irrespective of the chosen reference chart with a ‘personalised’ predictive model. An adequately powered prospective validation study in a different and diverse population is needed to externally validate the study findings. If the generalisability of the predictive models is so established, these predictive models could have significant clinical implications for managing and improving the outcome of dichorionic twin pregnancies. Even a predictive model incorporating only EFW and UA PI discordances could enhance risk stratification and facilitate early identification of dichorionic pregnancies at increased risk of stillbirth, especially those affected by sFGR. For such pregnancies, clinicians might consider more intensive monitoring protocols and scheduled birth based on the computed risk to enable a reduction in adverse pregnancy outcomes.

This study highlights the value of an integrated approach to risk prediction for adverse outcomes in dichorionic twin pregnancies. Combining inter‐twin EFW discordance with UA PI discordance has a high predictive accuracy for stillbirth or medically indicated ePTB for fetal indications. The management of dichorionic twin pregnancies is improved with such an approach compared to the current clinical convention of using arbitrarily dichotomous thresholds for either EFW discordance or SGA centile. After external validation, our prediction model could provide clinicians with a valuable tool for a more precise risk assessment with the potential to improve perinatal outcomes in dichorionic pregnancies through targeted intervention.

## Author Contributions

M.T., M.L., M.B., M.G.F. collected the data from the three centres; V.G. analysed the final dataset and wrote the first draft of the manuscript; A.B., J.C.J., D.A.B., and T.G. critically reviewed the manuscript; B.T., A.F., and E.B. planned the study, interpreted the results, and reviewed the first draft of the manuscript.

## Ethics Statement

Our study was conducted using fully anonymised retrospective clinical data, ensuring compliance with ethical and regulatory requirements across all participating centres. In the UK, the Health Research Authority (HRA) decision tool confirmed that NHS REC review was not required, as our study did not involve identifiable patient data, interventional procedures, or the disclosure of confidential data without consent. Under UK GDPR and the Data Protection Act 2018, anonymised data are not considered personal data, and similar principles apply under EU GDPR in Italy and Belgium. In both countries, retrospective studies using fully anonymised data do not require ethical approval unless mandated by national laws, which was not the case for our research. Accordingly, our study adhered to all relevant ethical and legal standards in the UK, Italy and Belgium.

## Conflicts of Interest

The authors declare no conflicts of interest.

## Supporting information


Data S1.

**Figure S1.** Cohort study flow chart. Created in https://BioRender.com.

**Figure S2.** Sensitivity and specificity of the prediction model including EFW discordance and UA PI discordance versus EFW discordance of 15%, 20% and 25%. CI: confidence interval, EFW: estimated foetal weight, UA PI: umbilical artery pulsatility index.

## Data Availability

The data that support the findings of this study are available from the corresponding author upon reasonable request.
