# Supplementary material for: Predicting Adverse Perinatal Outcomes in Dichorionic Twin Pregnancies: A Multicentre Cohort Study
Source: BJOG. 2025 Mar 7;132(7):983–90. doi: 10.1111/1471-0528.18125 (PMC12051245; doi:10.1111/1471-0528.18125)
Supplement: Supplementary file 1 — Data S1. [file BJO-132-983-s002.docx]

**Table S1.** Model intercepts, coefficients and calibration of the four models.

| **Model** | **Intercept** | **Coefficients** | **Hosmer-Lemeshow Test** |
| --- | --- | --- | --- |
| Model 1 | -5.824 | EFW discordance: 0.066 (p < 0.001) | Chi-square: 8.854 (p = 0.355) |
|  |  | UA discordance: 0.061 (p < 0.001) |  |
| Model 2 | -6.237 | EFW discordance: 0.080 (p < 0.001) | Chi-square: 3.822 (p = 0.873) |
|  |  | UA discordance: 0.070 (p < 0.001) |  |
|  |  | MCA PI discordance: 0.005 (p = 0.690) |  |
| Model 3 | -6.218 | EFW < 10th percentile: 1.379 (p = 0.011) | Chi-square: 5.836 (p = 0.666) |
|  |  | UA discordance: 0.080 (p < 0.001) |  |
| Model 4 | -6.635 | EFW < 10th percentile: 1.447 (p = 0.021) | Chi-square: 5.142 (p = 0.742) |
|  |  | UA discordance: 0.091 (p < 0.001) |  |
|  |  | MCA PI discordance: 0.010 (p = 0.346) |  |

**Table S2.** Prediction models for perinatal death (including stillbirth or neonatal death) and/or medically indicated preterm birth before 34 weeks gestation for fetal indications (n=74).

| Models | Variables | OR | 95% CI | AUC | 95% CI |
| --- | --- | --- | --- | --- | --- |
| 1 | - EFW discordance - UA PI discordance | 1.07  1.05 | 1.05-1.10  1.03-1.07 | 0.81 | 0.74-0.88 |
| 2 | - EFW discordance - UA PI discordance - MCA PI discordance | 1.09  1.07  0.99 | 1.06-1.12  1.04-1.09  0.97-1.02 | 0.87 | 0.81-0.93 |
| 3 | - SGA <10^th^ centile - UA PI discordance | 1.72  1.07 | 0.88-3.38  1.06-1.09 | 0.79 | 0.72-0.86 |
| 4 | - SGA <10^th^ centile - UA PI discordance - MCA PI discordance | 2.29  1.09  1.00 | 0.97-5.39  1.07-1.11  0.98-1.02 | 0.83 | 0.77-0.90 |

OR: odd ratio, CI: confidence interval, AUC: area under the curve. EFW: estimated fetal weight, UA: umbilical artery, PI: pulsatility index, MCA: middle cerebral artery,
