# Supplementary figures and images for: Predicting Adverse Perinatal Outcomes in Dichorionic Twin Pregnancies: A Multicentre Cohort Study
Source: BJOG. 2025 Mar 7;132(7):983–90. doi: 10.1111/1471-0528.18125 (PMC12051245; doi:10.1111/1471-0528.18125)

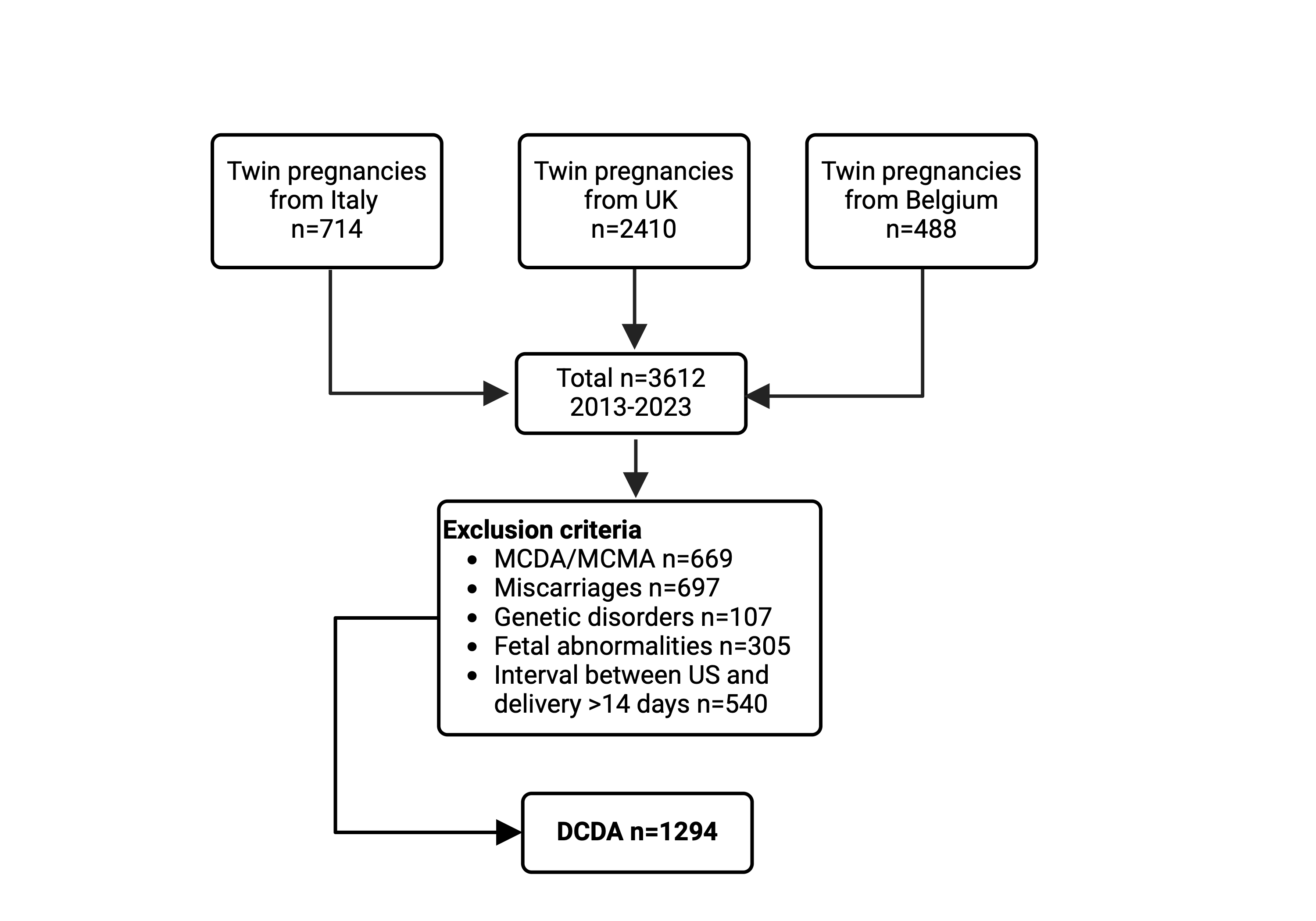

Supplement: Supplementary file 2 — Figure S1. Cohort study flow chart. Created in https://BioRender.com. [file BJO-132-983-s001.jpeg]

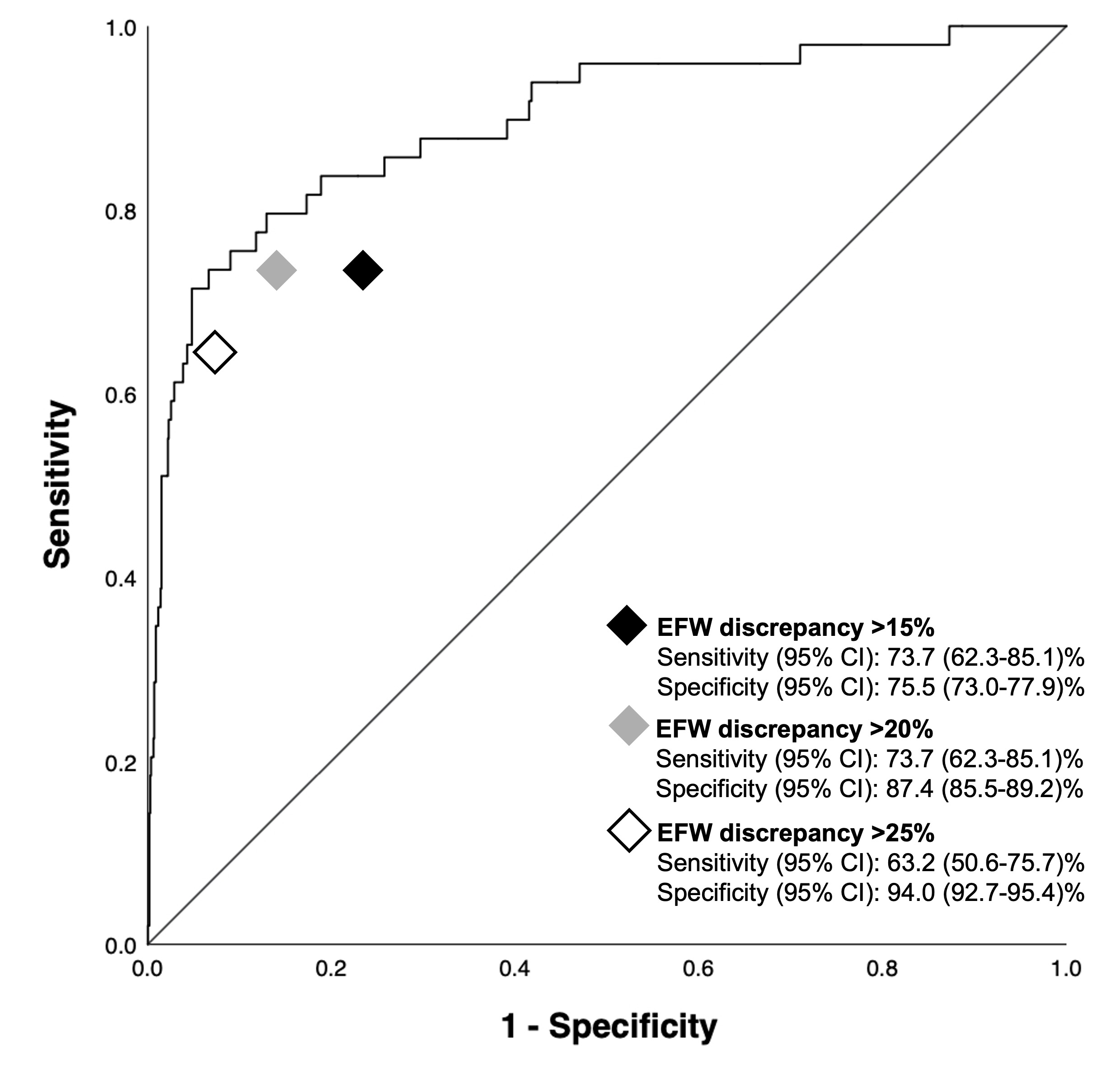

Supplement: Supplementary file 3 — Figure S2. Sensitivity and specificity of the prediction model including EFW discordance and UA PI discordance versus EFW discordance of 15%, 20% and 25%. CI: confidence interval, EFW: estimated foetal weight, UA PI: umbilical artery pulsatility index. [file BJO-132-983-s003.jpg]
